# Comparing Neuroimaging Processing Pipelines for a Study of Prenatal Stress and Early Childhood Brain Development

**DOI:** 10.51894/001c.143901

**Published:** 2025-09-30

**Authors:** Lakshmi Sravya Jayam, Ardaman Kaur, Martin Styner, Rebecca Knickmeyer

**Affiliations:** 1 College of Osteopathic Medicine Michigan State University

## 15

### BACKGROUND

Exposure to maternal psychosocial stress and environmental chemicals during gestation affects brain development, but underlying mechanisms remain poorly understood. The EnviroBrain study was established to investigate two key pathways that may link toxic exposures to disrupted brain structure and function: inflammation and the gut microbiome.

### OBJECTIVE

Develop a robust processing pipeline to investigate the impact of maternal psychosocial stress, environmental chemicals, inflammation, and the gut microbiome on subcortical volumes, cortical thickness, and surface area in children aged 4 to 6 years.

Methods: MRI scans were processed using FreeSurfer to segment the brain and quantify subcortical volumes, cortical thickness, and surface area. Two processing methods were compared: one using only T1-weighted scans and another incorporating both T1- and T2-weighted scans. Quality control assessments were conducted, and segmentations with significant over- or underestimations of cortical and subcortical volumes were excluded.

### RESULTS

Quality control assessments revealed no significant differences in segmentation quality between the two methods.

### CONCLUSION

Because the two processing methods generated similar results, we intend to use only T-weighted scans for the full-scale project. This should increase the number of children included in our final analysis as not all children are able to remain still through both a T1 and T2-weighted scan.

### FUTURE DIRECTIONS

We will investigate whether SynthSeg and SynthStrip, advanced FreeSurfer tools, enhance segmentation accuracy. By refining neuroimaging techniques, this research aims to improve our understanding of how early-life stressors shape brain development, ultimately informing interventions for at-risk children.

